# The (un)successful global spread of Acinetobacter baumannii ST3

**DOI:** 10.1099/mgen.0.001819

**Published:** 2026-09-15

**Authors:** Eradah Abu Sabah, Margaret M.C. Lam, Francois Lebreton, Patrick T. McGann, Mehrad Hamidian

**Affiliations:** 1 Australian Institute for Microbiology and Infection, University of Technology Sydney, Ultimo, NSW, Australia; 2 Department of Infectious Diseases, School of Translational Medicine, Monash University, Melbourne, VIC, Australia; 3 Multidrug-Resistant Organism Repository and Surveillance Network (MRSN), Diagnostics and Countermeasures Branch, CIDR, Walter Reed Army Institute of Research, Silver Spring, MD, USA

**Keywords:** *Acinetobacter baumannii*, antibiotic resistance, homologous recombination, plasmid, sequence type 3 (ST3)

## Abstract

*Acinetobacter baumannii* sequence type 3 (ST3) has been reported in multiple regions but has not achieved the global dominance of major clones such as ST1 and ST2. Here, we analysed 383 ST3 genomes, including 205 isolates from the Multidrug-Resistant Organism Repository and Surveillance Network (MRSN) and 178 publicly available genomes, to investigate the population structure, resistance gene repertoire and evolutionary dynamics of this lineage. Phylogenetic analysis revealed two major clades with multiple subclades, with 82% of genomes linked to the Middle East, highlighting this region as the likely origin or reservoir. Despite detection across 12 countries, ST3 remains relatively uncommon globally, except in Israel where it is locally abundant. Pan-genome analysis revealed a core genome comprising ~42% of total gene content, comparable to other major sequence types. Extensive recombination was observed, including large chromosomal segments, a total of ~350 kb and ~1.2 Mb shared with ST1 and ST2, respectively, as well as recurrent exchanges at the ISAba1-*ampC* locus. A chromosomal resistance island, designated AbST3GRI, was identified in nearly all genomes and exhibited structural diversity driven by IS*26*. A total of 132 genomes carried carbapenem resistance genes, predominantly *bla*_OXA-23_. However, ST3 genomes contain a relatively limited repertoire of resistance genes compared to globally dominant clones. Despite retaining most key virulence determinants, ST3 has not undergone widespread global expansion. Our findings suggest that, although genetically dynamic and capable of acquiring resistance, the lack of extensive antibiotic resistance gene repertoire may have limited the global success of this lineage.

**Impact Statement**

Investigating how *Acinetobacter baumannii* lineages become globally dominant while others remain regionally confined is important for better understanding the spread of antimicrobial resistance. This study provides the first comprehensive genomic analysis of sequence type 3 (ST3), a lineage that is widely distributed yet relatively uncommon compared to major global clones such as ST1 and ST2. We show that ST3 possesses key features typically associated with successful lineages, including the presence of major virulence determinants, the ability to acquire carbapenem resistance genes such as *bla*_OXA-23_ and a highly dynamic genome shaped by recombination and mobile genetic elements. Notably, ST3 genomes contain a conserved chromosomal resistance island (AbST3GRI) and exhibit evidence of gene exchange with other major clones. Despite these characteristics, ST3 has not achieved global dominance. Our findings indicate that, unlike highly successful clones, ST3 lacks a substantially enriched repertoire of antibiotic resistance genes. This suggests that the scale and diversity of resistance determinants, rather than their mere presence, may be a key factor driving the global success of *A. baumannii* lineages.

## Data Summary

All *Acinetobacter baumannii* sequence type 3 (ST3) genomes sequenced in this study are deposited in GenBank and available under the BioProject PRJNA1322037. GenBank accession numbers for publicly available ST3 genomes analysed in this study are also available in Table S1.

## Introduction

*Acinetobacter baumannii* is a Gram-negative opportunistic pathogen that causes a range of healthcare-associated infections including pneumonia, bloodstream, urinary tract and wound infections [1, 2]. Strains that cause infections are well known for having resistance against several antibiotics including carbapenems [1, 2]. In the late 1990s and early 2000s, epidemiological investigations across European hospitals led to the identification of three predominant multidrug-resistant clonal lineages, named European Clone I, European Clone II and European Clone III (ECIII), based on typing techniques available at the time (e.g. amplified fragment length polymorphism and PFGE) [3, 4]. These lineages, in particular clones I and II, were later renamed international clones 1 and 2 (IC1 and IC2) [5], or global clones 1 and 2 (GC1 and GC2) [6], respectively, as they were found to be globally disseminated and dominated other problematic clones [2, 7, 8]. However, among these diverse lineages, sequence type 3 (or ST3 as defined by the Institut Pasteur multi-locus sequence typing (MLST) scheme; also formerly known as ECIII) remained less prevalent than the more dominant ST1 and ST2 lineages [2, 5–8]. The earliest ST3 strains were first reported in Europe in the 1980s [3, 4], then occasionally reported in several countries including Lebanon, Iran, Israel, the Netherlands, Italy, Spain, the UK, the Czech Republic and Peru [9–20]. These were often associated with resistance to carbapenems and other major antibiotic classes [9–20].

Despite its clinical relevance and international distribution, ST3 remains under-characterized as its global epidemiology, resistance mechanisms, evolutionary dynamics and significance as a global clone was never comprehensively investigated. Here, we sequenced 124 ST3 strains recovered in US military facilities within the US (*n*=82) and Germany (*n*=42) (both US and Germany isolates were from military personnel medically evacuated from the Middle East, part of the MRSN surveillance programme). We studied the population structure, resistance evolution and genomic context of antibiotic resistance genes (ARGs) of these study isolates in addition to a global collection of 259 genomes of *A. baumannii* ST3 that were publicly available in GenBank (as of mid-2025).

## Methods

### Genomes studied

A total of 383 ST3 *A. baumannii* genomes were analysed, including 124 genomes that were sequenced in this study (Table S1). The newly sequenced genomes (*n*=124) originated from the MRSN surveillance programme which targets MDR organisms (as defined by Magiorakos *et al*. [21]) from across the US Military Health System, including from soldiers injured and medically evacuated during Operation Iraqi Freedom (OIF) and Operation Enduring Freedom (OEF). Between 2003 and 2025, 8,090 *A*. *baumannii* isolates from 6,652 patients were collected and sequenced, and this collection was used to identify ST3 genomes. In addition to the genomes included in this study, the MRSN collected a total of 3 ST3 isolates between 2003 and 2025, all from Peru. However, further approval, from the sender, to include these three genomes was not sought, and these were excluded from further analysis.

### Whole-genome sequencing and genome assembly

Genomic DNA from the 124 strains was subjected to sequencing on an Illumina MiSeq platform, producing 150 bp paired-end reads. Quality control of the raw sequencing data was performed using FastQC [22] followed by trimming of adaptors using Btrim v0.2.0 [23]. Newbler (https://github.com/etheleon/newbler) was used to assemble short reads into draft genomes. The genome sequence of two representative ST3 genomes was also completed using the combination of short- and long-read (MinION) sequence data. Long-read sequencing was done using the R10.4.1 flow cell. Basecalling was conducted with Dorado v9.0.0 (https://github.com/nanoporetech/dorado/) using the super-accurate model (dna_r10.4.1_e8.2_400bps_sup@v5.0.0). Complete genome assemblies were generated using Autocycler v0.2.0 (https://github.com/rrwick/Autocycler/tree/v0.2.0). The resulting assemblies were further polished using Illumina short-read data with Polypolish (https://github.com/rrwick/polypolish). The quality (i.e. completeness and contamination) of all genomes including those downloaded from NCBI and those we sequenced and assembled in this study were examined using the CheckM v1.2.3 software (available at https://github.com/Ecogenomics/CheckM) [24].

### Sequence analysis

Genomes were annotated using Prokka v1.13.4 [25]. ARGs were detected using ABRicate v0.8.13 [26]. Mobile genetic elements were detected using ISFinder [27] and standalone blast v2.16.0 [28]. Protein-coding regions of plasmids and genomic resistance islands were annotated using the blastp v2.16.0 [28] and UniProt searches [29]. The mlst v2.16.1 programme [30] was used to confirm the sequence types (using both the Institut Pasteur and Oxford MLST schemes).

cgMLST (core genome Multi-Locus Sequence Type) was assigned using the SeqSphere+ software (https://www.ridom.de/seqsphere/ug/v40/Overview.html) and the *A. baumannii* cgMLST scheme developed by Higgins *et al*. [31]. Genomes were included only if they contained at least 90% of the 2,390 target genes in the cgMLST scheme. Kaptive v3.0 [32] was used to identify the capsular polysaccharide (KL) and lipo-oligosaccharide outer core (OCL) synthesis loci.

Plasmid replication initiation genes (*rep*/Rep types) were determined using the *Acinetobacter* Plasmid Typing scheme [33]. Genomic and plasmid structure figures were drawn to scale using the SnapGene*®* v6.0.5 software and Illustrator*®* v26.2.1.

Virulence-associated determinants were identified using a manually curated local database of virulence genes (e.g. genes involved in iron acquisition, biofilm formation, capsule biosynthesis and regulatory systems) as previously described [34].

### Core genome and phylogenetic analysis

Pan- and core-genome analyses were performed using the Panaroo software [35]. For each sequence type, we computed the core and accessory gene sets. For comparison purposes, a set of representative ST1 (*n*=190 genomes) and ST2 (*n*=121 genomes), including the earliest and latest genome from each country, as well as all available ST15 (*n*=194 genomes) and ST25 (*n*=542 genomes) were compiled. These sequence types were selected as they represent some of the most epidemiologically important and globally distributed clones. Core-genome sizes were then compared among the five sequence types (i.e. ST1, ST2, ST3, ST15 and ST25) to investigate differences in genomic diversity. The maximum-likelihood (ML) phylogeny was reconstructed from the Panaroo v1.3 [35] core-genome alignment and rooted with the *A. baumannii* A1 (ST1) outgroup (GenBank accession no. CP010781). Recombination filtering was subsequently performed using Gubbins v3.4 [36] to obtain a high-quality, recombination-free core-genome SNP alignment. For temporal analysis, a separate recombination-free SNP alignment was generated using RedDog (https://github.com/katholt/RedDog) and filtered using Gubbins v3.4. An ML phylogeny was reconstructed using IQ-TREE v2.3.6 (https://github.com/iqtree/iqtree3). Eleven isolates (Ab175, Ab166, F370, D297, D255, ARC7086, TIDB3552, 18A2229, 2024BM-00103, SUD-IS-P1 and 2024BM-00198) were excluded due to unstable and biologically implausible branch lengths, likely resulting from sequencing errors that disrupted the molecular clock signal. For the root-to-tip regression analysis, the resulting recombination-free ML tree and SNP alignment were further analysed using TreeTime v0.9.4 (https://github.com/neherlab/treetime) under a relaxed molecular clock using the General Time Reversible substitution model. Ten independent date-randomization analyses and the clock-filter 3 option were used to optimize branch lengths and estimate divergence times as we previously described [37]. Eleven genomes were identified as temporal outliers in the regression analysis and were excluded from the temporal analysis.

All phylogenetic trees were visualized and annotated in R using ggplot2 v3.5.1 [38] within RStudio [39]. Antibiotic resistance genes (ARGs), recombination blocks and other relevant metadata were mapped onto each tree and visualised in RStudio.

## Results and discussion

### Genome dataset, general properties and geographical distribution

In this study, we analysed 383 ST3 *A. baumannii* genomes, including 205 genomes from the Multidrug-Resistant Organism Repository and Surveillance Network (MRSN), 124 of which were sequenced in this study and 81 MRSN genomes were already available in GenBank. Our dataset also included an additional 178 genomes available in GenBank as of mid-2025, mainly sampled from non-military sources across various geographical regions. All MRSN genomes were from strains isolated at US Military Treatment Facilities, primarily originating from Middle Eastern battle zones but recovered after medical evacuation to the USA (*n*=163 isolates) or US military hospital in Germany (*n*=42 isolates). Here, we also completed the genomes of two MRSN strains (MRSN7088 and MRSN11709; Table 1), which were selected as representatives of the two major ST3 phylogenetic clades. Their genomes were ~4 Mb in size, which is similar to the size range in ST1 [37] and ST2 [40] genomes.

**Table 1.** Properties of complete ST3 genomes

Strain	Size (bp)	Plasmid Rep	Mob	Acquired resistance gene	Additional important plasmid encoded function	GenBank no.
**MRSN7088**	3,983,691	na*	na	*aadB, tetA, sul1, aadA1, strA/B, aphA6*	na	JBQYLC010000001
p1MRSN7088	6,078	–		*aadB*	–	JBQYLC010000002
p2MRSN7088	30,183	R3-T12, R3-T33, R3-T203	Mob_Q_	*tetA, aphA1*	Toxin and antitoxin, DNA replication and repair, binds DNA	JBQYLC010000003
p3MRSN7088	122,469	–	na	*strA/B, sul2, tetA*	T4SS conjugation, DNA repair, transcriptional regulation	JBQYLC010000004
**MRSN11709**	4,000,016	na	na	*aadB, tetA/R, sul1, aadA1, strA/B, aphA6*	na	JBQYLB010000001
p1MRSN11709	26,094	R3-T35, R3-T12	–	–	Toxin and antitoxin, DNA replication and repair, DNA recombination and repair	JBQYLB010000002
p2MRSN11709	32,825	R3-T23, R3-T33, R3-T203	Mob_Q_	*tetA, aphA1*	Toxin and antitoxin, DNA replication and repair, DNA binding, vitamin B12 uptake, heme degradation	JBQYLB010000003

*Not applicable

**Fig. 1.**
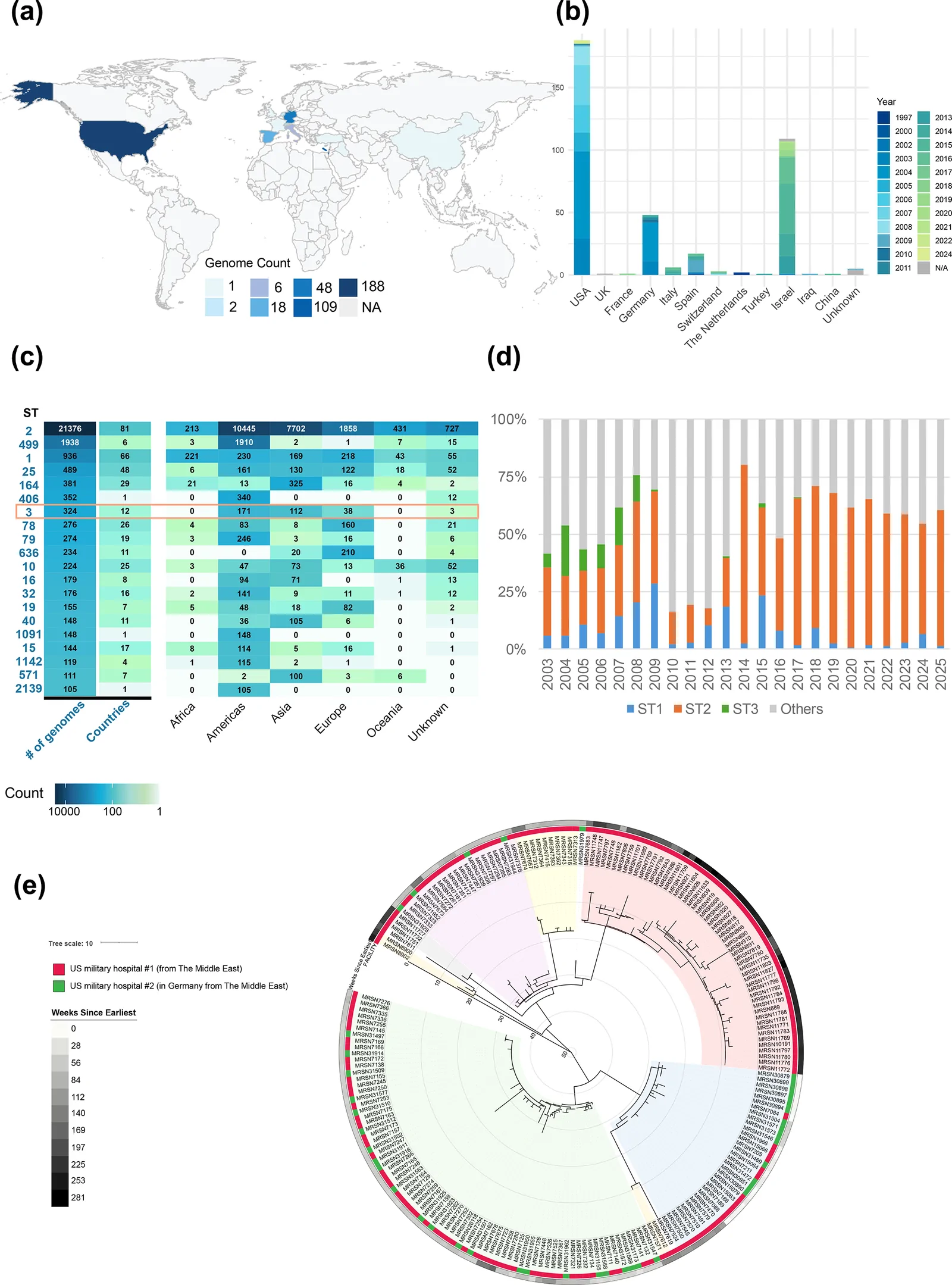
Global distribution of *A. baumannii* ST3 isolates. (**a**) Geographic distribution of ST3 isolates. (**b**) Collection dates of isolates or genomes. (**c**) Distribution of the top 20 STs across different continents. (**d**) Prevalence of ST3 in MRSN *A. baumannii* collection (*n*=6,652 deduplicated genomes) from 2003 to 2025. (**e**) Phylogenetic tree based on cgMLST distance matrix for MRSN ST3 isolates.

The ST3 genomes were identified from 12 countries, predominantly from the USA (*n*=188), Germany (*n*=48) and Israel (*n*=109) but also the UK, France, Italy, Spain, Switzerland, the Netherlands, Turkey, Iraq and China (Fig. 1a, b), demonstrating a wide geographical spread. Notably, among the genomes from the USA and Germany, only 25 and 6, respectively, were non-MRSN. Despite early reports of ST3 from several European countries [4, 20], 82.5% (*n*=316) of genomes were either from the Middle Eastern countries or linked to the region (i.e. MRSN strains exported to other regions), suggesting that this lineage likely originated or was established there. Despite early reports of ST3 in European countries, no recent genomes were observed in GenBank, likely due to their low abundance in Europe and dominance of other clones in the region. This is further supported by analysis of 6,652 MRSN isolates recovered between 2003 and 2025, which showed that the ST3 isolation peaked between 2003 and 2008 and was rarely detected thereafter (Fig. 1d).

Thus, to gain insights into the global distribution of ST3s in the context of other major sequence types, we analysed the geographical distribution of the 20 most prevalent sequence types in GenBank (Fig. 1c). While ST3 genomes accounted for the seventh most prevalent ST, their spread appeared to be limited to certain regions compared to the wider geographical spread of other prevalent sequence types (Fig. 1b, c). Similarly, ST499 and ST406 also appear to be limited to the Americas, whereas the well-known ST1, ST2 and ST25 clones show more globally distributed patterns (Fig. 1c). To investigate this further, we examined the proportion of ST3 genomes relative to other genomes across the 12 countries in which ST3 was detected (Table S2). Notably, except for Israel, where ST3 genomes accounted for 35.5% of all available genomes, the proportion of ST3 in other countries ranged from 0.02% (China) to 4.55% (the Netherlands), indicating that, despite their spread to 12 countries, ST3 remains relatively uncommon in most countries.

### Phylogenetic relationships, pan-genome structure ARGs and temporal evolution

**Fig. 2.**
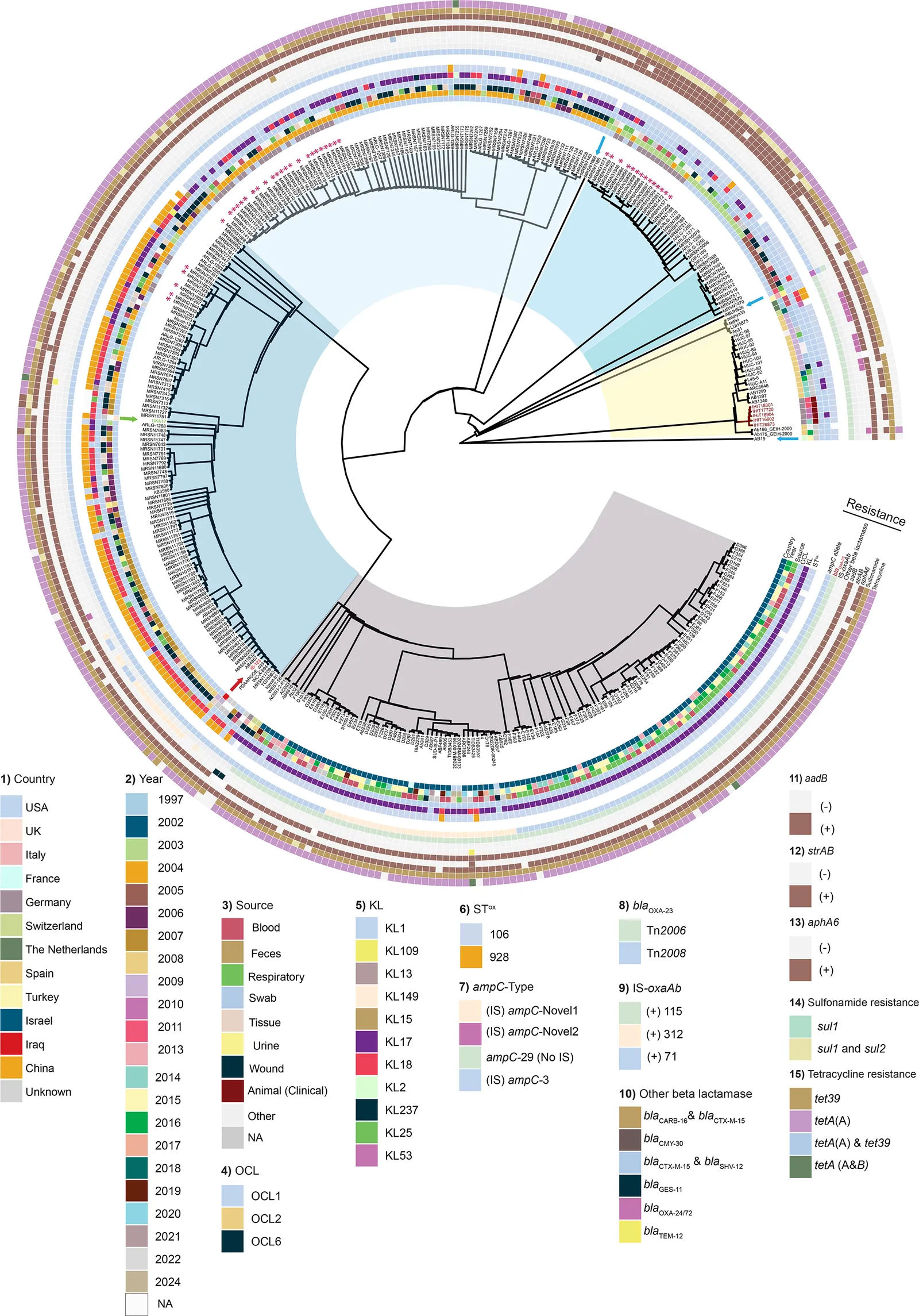
ML phylogenetic tree of ST3 *A. baumannii* isolates. The tree is generated from recombination-filtered core-genome SNPs. Metadata, including country, year of isolation, source, KL and OCL types, *ampC* alleles and resistance genes, is shown on different rings around the tree. ‘NK’ indicates ‘not known’. Distinct clades and subclades are shaded in different colours, with subclade designations indicated inside the shaded areas. Colour coded key is shown.

We used our dataset to reconstruct the population structure of this lineage and characterize its pan-genome composition. From the ML phylogenetic tree (constructed using SNPs not associated with recombination events) (Fig. 2), ST3 genomes separated into two main clades comprising five subclades, each containing 10 to >80 genomes (Fig. 2). The MRSN genomes were distributed across four distinct subclades and, except for two non-MRSN, non-military isolates (IS-123 and UKA17; indicated by green and red arrows in Fig. 2), no other strains clustered closely with them. IS-123 is a clinical isolate recovered in Iraq, consistent with its phylogenetic proximity to the MRSN genomes. UKA17, recovered in the UK, likely represents an imported strain originating from the Middle East. Notably, the ST3 dataset was predominantly composed of human-associated isolates, with five equine uterine biopsy isolates also identified (Fig. 2). Although ST3 has been reported from non-human sources, including animals [41, 42], genomic representatives from non-human sources remain limited. Further, all genomes from Israel (*n*=109) clustered together within one of the main clades (marked in grey in Fig. 2), indicating the homogeneity of the local strains circulating in the country. All European genomes appear to be distinct from the military isolates and those from Israel regardless of their isolation date, indicating early divergence. Moreover, all MRSN genomes from Germany (marked by an asterisk in Fig. 2) tightly clustered with the rest of the MRSN group from the Middle East, confirming their shared isolation source and link to the region. cgMLST analysis of our MRSN genomes revealed several clear clusters, consistent with the recombination-free tree, indicating outbreaks within the military genomes set (Fig. 1e).

**Fig. 3.**
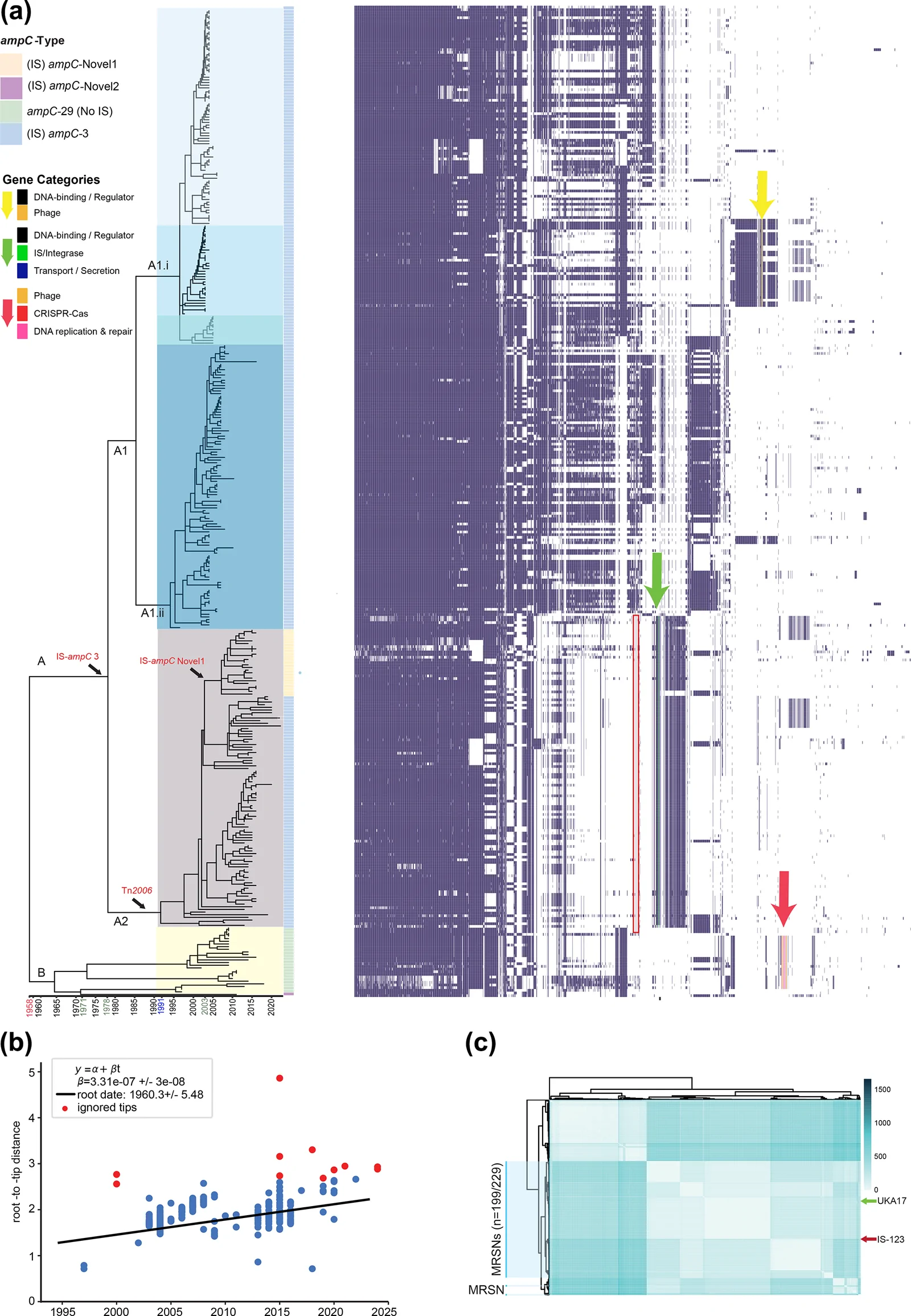
Dated phylogenetic tree and pan-genome structure of the global ST3 dataset. (**a**) Accessory genome clusters and core-genome proportion in ST3 plotted against the dated phylogenetic tree with the years indicated below the tree. (**b**) Root-to-tip regression of genetic distance against year of isolation (*R*^2^=0.37; red dots indicate genomes that were excluded due to signal noise). (**c**) SNP distribution and clusters across the ST3 set. Histogram showing the number of SNPs detected among ST3 isolates. The arrows indicates two isolates that are not part of the MRSN collection but fall within the MRSN branches.

**Fig. 4.**
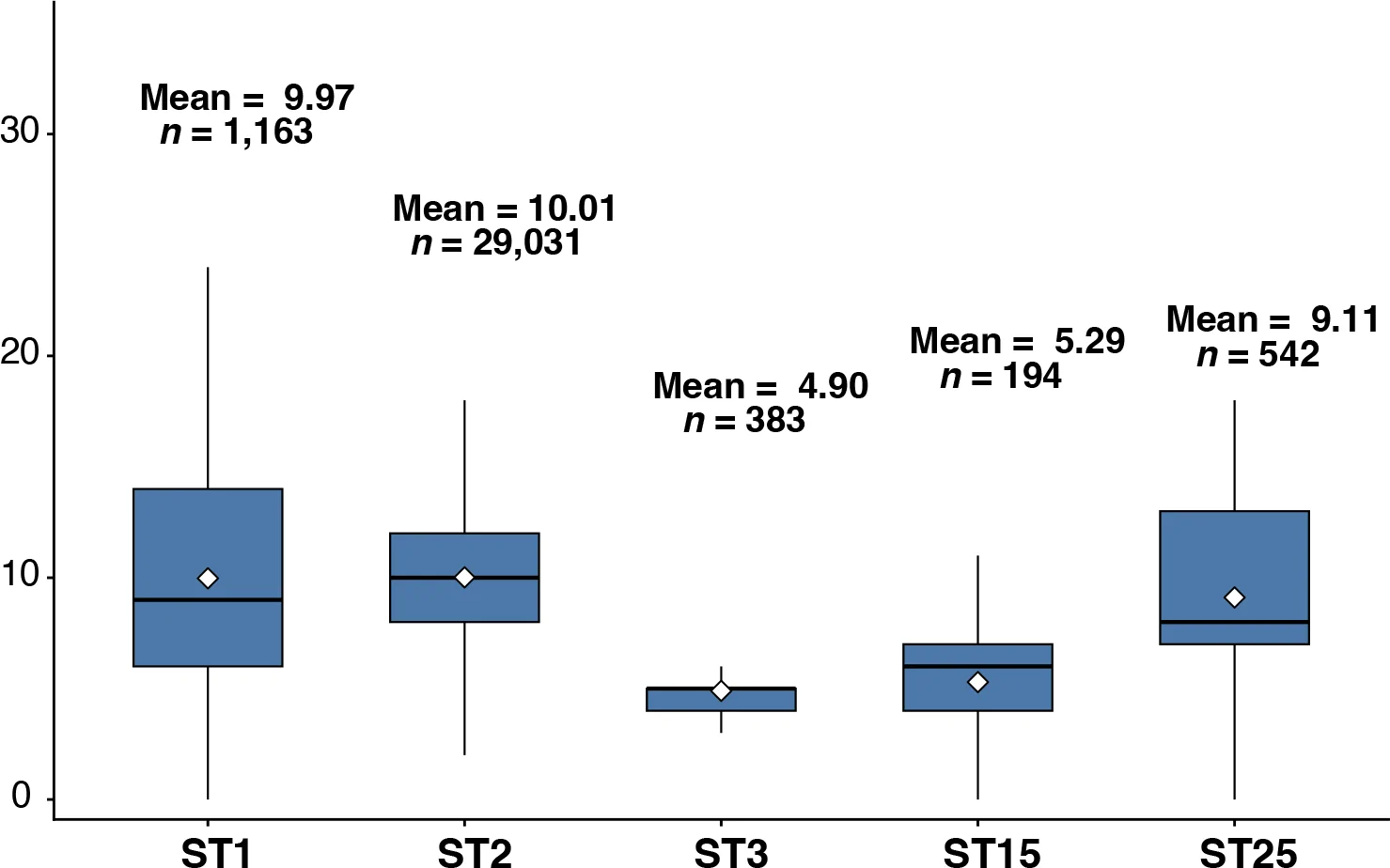
Boxplots showing the variation in the number of acquired ARGs in all publicly available ST3 compared to ST1, ST2, ST15 and ST25 genomes.

Considerable variation was observed in the surface polysaccharides (i.e. OC and K loci; Fig. 2) and ARGs of ST3 genomes. For example, 132/383 genomes contained a carbapenemase, including 120 carrying *bla*_OXA-23_ and 12 carrying *bla*_OXA-24_. Notably, all but 11 of the *bla*_OXA-23_ containing genomes were from Israel (Fig. 2). In contrast, the 12 genomes carrying *bla*_OXA-24_ were derived from non-military Spanish isolates (11 genomes) and USA (1 non-military clinical sample) and none from either the MRSN collection or Israeli genomes (Fig. 2). All ST3 genomes, with the exception of European non-military genomes (marked yellow in Fig. 2), included an ISAba1 upstream of the *ampC* gene (Fig. 3), accounting for resistance to third-generation cephalosporins [1, 43]. Several other ARGs were also common in this dataset, including the *aadB* gene conferring resistance to tobramycin, kanamycin and gentamicin; *tetA*(A) tetracycline resistance gene; and *aphA6* amikacin resistance gene (Fig. 2) . All ST3 genomes contained at least one acquired ARG. However, compared to the rich ARG repertoires often observed in other globally distributed clones, such as ST1, ST2 or ST25 [1], ST3 does not appear to carry a substantial ARG content overall. To quantify and compare the resistome across major globally distributed lineages, we quantified the number of acquired ARGs per genome. ST3 carried substantially fewer ARGs (mean=4.90; *n*=383) than ST1 (mean=9.97; *n*=1,163), ST2 (mean=10.01; *n*=29,031) and ST25 (mean=9.11; *n*=542) and was comparable to ST15 (mean=5.29; *n*=194) (Fig. 4). Notably, up to four of the ARGs identified in ST3 are frequently located within the AbST3GRI chromosomal genomic resistance island, highlighting its contribution to the otherwise limited resistome of this lineage.

Our analysis revealed a core genome comprising ~42% (Table 2) of the total gene content, with the majority of accessory genes clustering into four major groups (A1.i, A1.ii, A2 and B in Fig. 3a). To compare this with other major sequence types, we surveyed the literature and found that reported core and accessory genome sizes vary widely (e.g. core genomes ranging from 1.7% to 70%; Table 2), largely due to inconsistent inclusion of diverse, and in some cases very closely related, sets of genomes. To analyse this further, we curated representative genome sets for ST1 (190 genomes), ST2 (121 genomes), ST15 (194 genomes) and ST25 (542 genomes) (Table 2), with each set comprising representatives of the main lineages identified through phylogenetic analysis. Using this approach, we showed that the ST3 core genome is comparable to those of ST2 (44.06%) and ST15 (38.4%), whereas ST1 and ST25 include a smaller core genome (14.94% and 20.8%, respectively), indicating a more diverse genomic structure.

**Table 2.** Pan-genome characteristics of diverse *A. baumannii*

Genus/species	ST	No. of genomes	Pan genome*	Core genome†	Soft core genome	Accessory genome	Reference
*A. baumannii*	ST2	121	5,839	2,572 (44.06%)	594	2,673	This study
*A. baumannii*	ST1	190	6,562	981 (14.94%)	1,228	4,353	This study
*A. baumannii*	ST3	383	5,174	2,197 (42.45%)	1,080	1,897	This study
*A. baumannii*	ST25	542	11,041	2,295 (20.8%)	545	8,201	This study
*A. baumannii*	ST15	194	6,096	2,340 (38.4%)	534	3,222	This study
*Acinetobacter*	15 species	312	47,500	818 (1.72%)	–	22,291	[54]
*A. baumannii*		2,112	19,000	2,353 (12.38%)	322	2,968	[55]
*A. baumannii*	23 STs	249	11,694	1,867 (15.96%)	2,833	10,602	[56]
*A. baumannii*	22 STs	78	7,683	1,344 (17.49%)	–	4,644	[57]
*A. baumannii*	6 STs	35	4,036	2,830 (70.12%)	–	1,206	[58]
*A. baumannii*	8 STs	18	5,009	2,007 (40.08%)	–	3,002	[59]

*Numbers indicate the number of genes identified under pan, core, soft core and accessory genomes.

†% shows the proportion of core genes among all detected genes.

Accessory genome analysis revealed patterns consistent with numerous major gene acquisition events (Fig. 3a, c). A closer examination of nine of these loci showed that they primarily contain horizontally acquired elements, including regions encoding insertion sequence transposases, CRISPR-Cas systems, prophage regions and other functions such as DNA replication (likely reflecting specific plasmid acquisition) or DNA repair systems (yellow, green and red vertical arrows in Fig. 3). Root-to-tip regression analysis revealed a moderate temporal signal (*R*^2^ = 0.37), consistent with a measurable accumulation of substitutions over time. Time-scaled phylogenetic reconstruction using TreeTime estimated an evolutionary rate of 3.3×10⁻⁷ substitutions site⁻¹ year⁻¹ and dated the most recent common ancestor of the lineage to ~1958 (Fig. 3b). In a similar study, we previously showed a 1.5×10^−6^ substitutions site⁻¹ year⁻¹ for a set of ST1 genomes recovered over 50 years [37], which is ~22% higher than observed in ST3.

### Evidence for chromosomal exchange between ST3 and major global clones (ST1 and ST2)

**Fig. 5.**
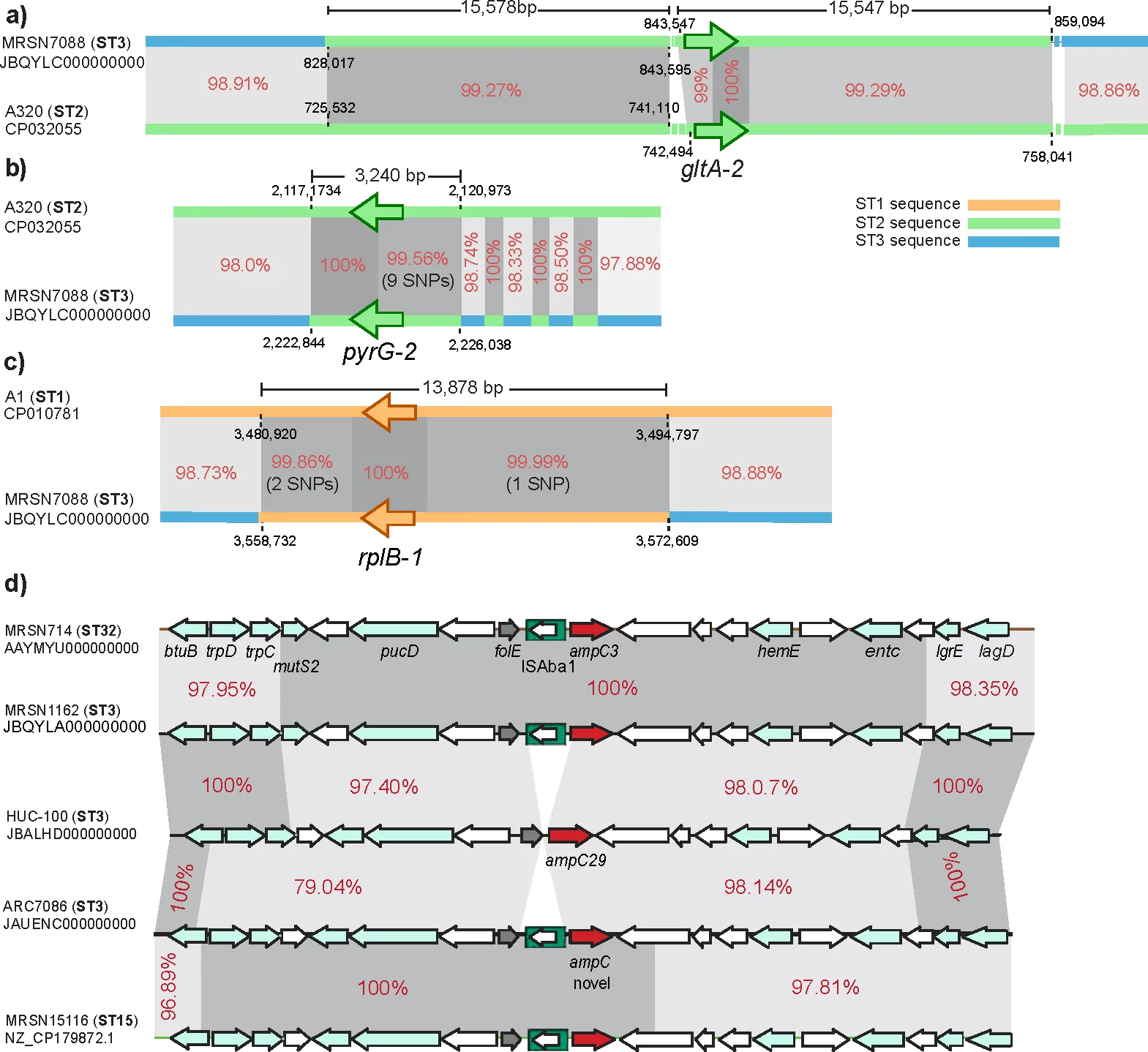
Comparative analysis of MLST loci in ST3. (**a**) Alignment of the* gltA* genomic region between ST3 isolate MRSN7088 and ST2 isolate A320 (CP032055). Numbers labelled on grey shadings indicate % identity. (**b**) Alignment of the* pyrG* locus between ST3 isolate MRSN7088 and ST2 isolate A320 (CP032055). (**c**) Comparison of the* rplB* locus between representatives of ST3, ST1 and ST2. Numbers shown along the sequences represent chromosomal coordinates of the corresponding genomic regions. (**d**) Schematic representation of the chromosomal *ampC* region in ST3 genomes. The *ampC* gene is highlighted with a red arrow, and ISAba1 insertions are shown as green boxes. Grey shading indicates regions of nucleotide identity, with percentage identities labelled in red.

*A. baumannii* strains are known to incorporate large segments of DNA via homologous recombination, a phenomenon that appears to play a significant role in the acquisition of genetic material, leading to genomic evolution and the emergence of new lineages [1, 44, 45]. ST3 includes the *cpn60-3*, *fusA-3, gltA-2, pyrG-2, recA-3, rplB-1* and *rpoB-3* allelic profile in the Institut Pasteur MLST scheme. We noted that two alleles (*pyrG-2* and *gltA-2*) are identical to those in ST2, and one allele (*rplB-1*) is identical to the *rplB* allele present in ST1 genomes, suggesting potential incorporation of DNA segments and likely co-evolution with ST1 and ST2. Thus, we examined chromosomal regions potentially imported through recombination, including those surrounding *gltA-2, pyrG-2* and *rplB-1* and identified 31, 3.2 and 13.8 kb recombinant segments, respectively, potentially acquired from ST2 and ST1 genomes (Fig. 5).

To identify additional DNA segments potentially obtained from ST1 and ST2, we compared the core genomes of our curated datasets of ST1 (*n*=190 genomes), ST2 (*n*=121) and ST3 (*n*=383). Detailed comparative genomic analysis (using blast) identified dozens of regions within the ST3 chromosome, ranging in size from less than 1 kb to over 50 kb, that share high identity (i.e. 99.9–100%) with corresponding loci in ST1 and ST2. Overall, ST3 genomes share ~9.2% of their genomes with ST1 (~350 kb in total, with 100% identity) and 27.8% with ST2 (equivalent to ~1.2 Mb in total), implying potential historical recombination or gene flow events between ST1, ST2 and ST3. Although the direction of gene flow is difficult to determine, such exchanges may have contributed to the diversification of ST3, and possibly also ST1 and ST2. The identification of these chromosomal regions highlights the dynamic nature of genome evolution in *Acinetobacter* and the potential for genetic interactions between distinct lineages.

Other notable recombination events include multiple exchanges in the ISAba1-*ampC* region. We previously provided evidence for the exchange of this region between ST3 genomes and ST32 and ST15 lineages [44, 46]. Here, we analysed the full ST3 dataset and show that clade B (comprised mainly of European non-military strains; highlighted in yellow in Figs 2 and 3) includes the original *ampC* sequence, with no ISAba1 preceding the gene (for example HUC-100 in Fig. 5d). However, with the exception of one Israeli sub-lineage (*n*=33) and one USA singleton carrying a novel *ampC* sequence, the remaining genomes (*n*=320) contain a 15,984 bp recombinant DNA segment that includes the ISAba1-*ampC* structure (MRSN1162 in Fig. 5d). We previously identified this segment in a subset of ST3 genomes and showed that it was acquired from ST32 genomes [46]. Here, we confirm its presence in the majority (*n*=320) of ST3 genomes across multiple clades, indicating acquisition in an early ancestor.

We also analysed the genomic region surrounding the *ampC* gene in the Israeli subclade and identified an additional 19,690 bp recombinant DNA segment (ARC7086 in Fig. 5d). Notably, this segment is also shared by a subset of ST15 [44] and ST79 genomes. Detailed searches of GenBank and draft genome collections did not identify a likely source, suggesting that this segment originates from a yet uncharacterized sequence type. Together, these indicate the extensive and recurrent recombination at the *ampC* locus and its role in multiple sequence types including ST3.

### Diversity and origins of chromosomal resistance islands carrying aminoglycoside, tetracycline and sulphonamide resistance genes

**Fig. 6.**
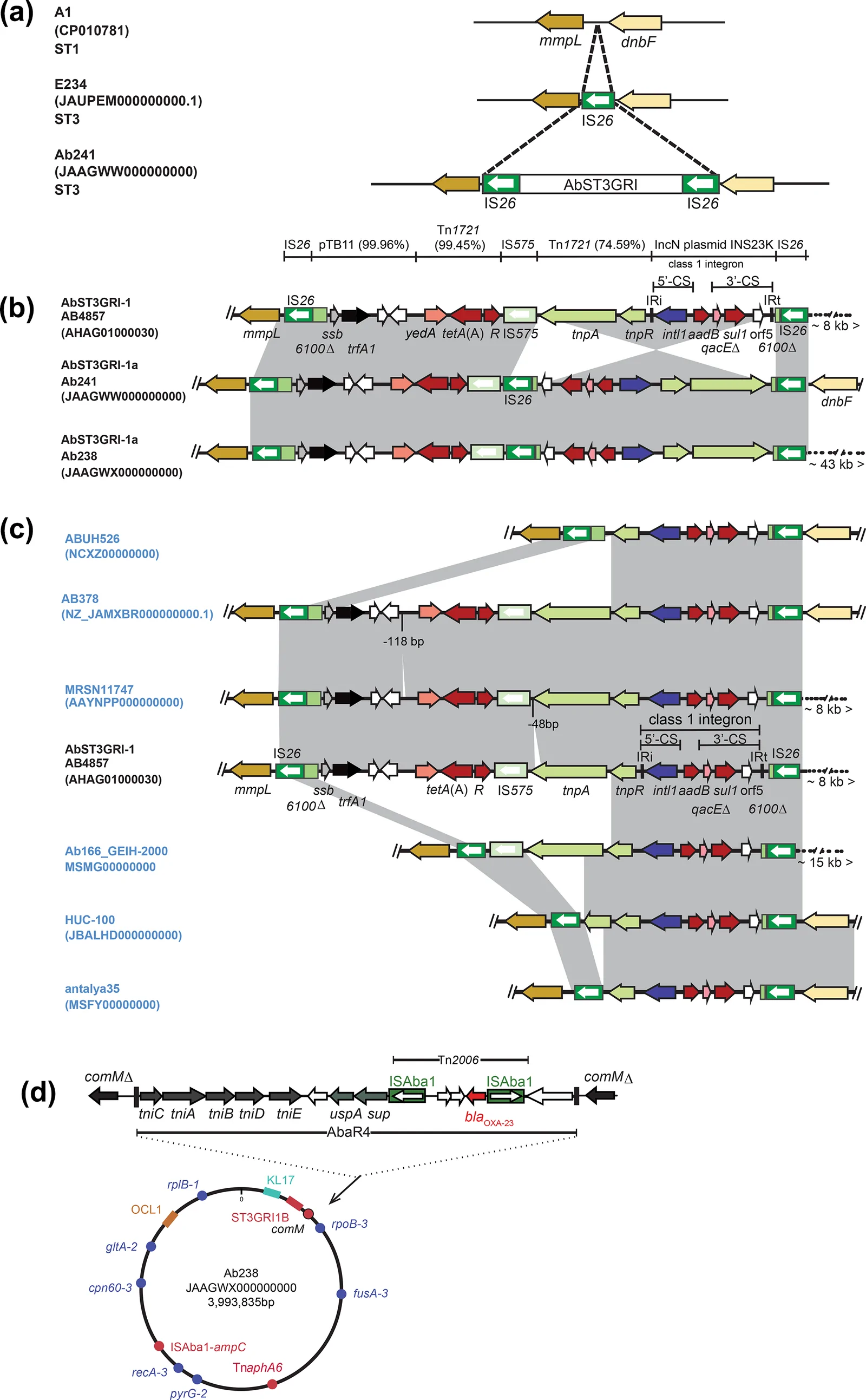
Structure of resistance islands identified in ST3 genome. (**a–c**) Schematic comparison of the different AbST3GRI genomic resistance island and its variants characterized in ST3 genomes. Each panel represents a distinct variant, with genes drawn as arrows. Antibiotic-resistance genes are coloured red and insertion sequences (e.g. IS*26*) shown as green boxes. Class 1 integrons and IS*26*-flanked segments are indicated, and regions of nucleotide identity. (**d**) Structure of the AbaR4 resistance island and its chromosomal position (in the *comM* gene) in *A. baumannii* Ab238 (ST3). The chromosomal layout of strain Ab238 (JAAGWX000000000; 3,993,835 bp) is shown, with Pasteur MLST allele numbers mapped to their genomic positions.

Karah *et al*. previously reported a single ST3 strain (strain A085, recovered in Israel in 2013) that carries the *aadB* gene on a class 1 integron located within an 18,625 bp chromosomal structure [16]. Here, we analysed this region in detail and screened our ST3 dataset to determine the presence of this putative genomic island, or its variants, in ST3 genomes. Notably, except for five genomes, our analyses predicted a variant of this region in all ST3 chromosomes. A copy of IS*26* was detected at the insertion point (between the *mmpL* and *dnbF* genes; at bases 3,763,942–3) in all five ST3 genomes lacking the genomic island (e.g., E234; Fig. 6a), indicating that this region was likely assembled and incorporated into the ST3 chromosome via IS*26*. However, this intergenic region is intact in all ST1 genomes, including A1 (GenBank no. CP010781), indicating the ST3-specific nature of this acquisition (Fig. 6a).

The largest structure measures 19,453 bp and carries three ARGs: *aadB* (tobramycin, kanamycin and gentamicin), *tetA*(A) (tetracycline) and *sul1* (sulphonamide). Given the presence of features consistent with a genomic resistance island and its exclusive presence in ST3 genomes, we designated this region the *A. baumannii* ST3 genomic resistance island (AbST3GRI). We tracked different segments of AbST3GRI and identified their origins, which include several known transposons (e.g. Tn*1721*, carrying *tetA*(A) and plasmids). An intact copy of the AbST3GRI class 1 integron was also found in INS23K (GenBank accession no. EU880929.1), a 64 kb IncN plasmid from *Klebsiella pneumoniae*. In total, nine variants of AbST3GRI were identified, mainly arising from IS*26* insertions or IS*26*-internal or -adjacent deletions, some extending into the chromosome (Table 3, Fig. 6b, c). The majority of genomes carried either AbST3GRI-1 or AbST3GRI-1a (i.e. AbST3GRI-1::IS*26*). These highlight the central role of IS*26* in generating structural diversity and chromosomal integration of resistance islands in ST3 genomes.

**Table 3.** Properties of AbST3GRI variants

AbST3GRI variant (strain)	No. of strains	Resistant genes	AbST3GRI length (bp)	Internal deletion (bp)	IS*26-*mediated chromosomal deletion
1 (AB4857)	228	*tetA*(A)*, aadB*, *sul1*	18,625	0	8,154
1a (Ab241)	122	*tetA*(A)*, aadB*, *sul1*	19,453	0	0
1a (Ab238)	2	*tetA*(A)*, aadB*, *sul1*	19,453	0	43,065
2 (AB378)	2	*tetA*(A)*, aadB*, *sul1*	18,407	118	0
3 (MRSN11747)	2	*tetA*(A)*, aadB*, *sul1*	18,577	48	8,068
4 (Ab166_GEH-200)	2	*aadB*, *sul1*	8,345	10,280	15,753
5 (HUC_100)	16	*aadB*, *sul1*	7,737	10,912	0
6 (ABUH526)	3	*aadB*, *sul1*	5,974	11,855	0
7 (antalya35)	1	*aadB*, *sul1*	5,701	12,121	0

### AbaR4 carbapenem resistance island found in the chromosomal *comM* gene

In addition to the ARGs in AbST3GRI (*tetA*(A), *aadB* and *sul1*), 132 genomes carried carbapenem resistance genes. The *bla*_OXA-24_ gene (*n*=12 ST3 genomes) was associated with <40 kb R3-type plasmids as reported previously [1, 2]. In contrast, *bla*_OXA-23_ (*n*=120) was found within Tn*2006* or Tn*2008*, in a range of genomic contexts including both chromosomal and plasmid locations; these transposons are known to drive the dissemination of this gene [1]. In two ST3 genomes, both from Israel (strains Ab238 and Ab241), *bla*_OXA-23_ was in Tn*2006*, which was inserted within the AbaR4 island in the *comM* gene (Fig. 6d). AbaR4 is a 16 kb genomic island with a backbone (Tn*6022*) related to AbaR-type genomic resistance islands, which are frequently found in the chromosomal *comM* gene of ST1 strains. In 2011, we described D36, an ST81 strain (belonging to global clone 1, GC1) carrying AbaR4 in the chromosomal *comM* gene, replacing an AbaR3-type island [47]. Later, we reported an ST2 strain (Cl415, recovered in 2017 in Lebanon) with AbaR4 in *comM*, a site more commonly occupied by variants of AbGRI1 genomic resistance islands [48]. These findings add ST3 to the sequence types known to carry AbaR4 in the *comM* gene and further highlight this gene as a hotspot for integration.

### Genetic structure of plasmids carried by MRSN7088 and MRSN11709 and distribution of plasmid replication genes across ST3

*A. baumannii* harbours a diverse repertoire of plasmids that are widely recognised as one of the drivers of AMR evolution and spread [33, 49–51]. Thus, to better understand the structure of plasmids in MRSN7088 and MRSN11709, we performed a comparative analysis with related plasmids found in GenBank to track the origin. MRSN7088 contained three plasmids, and MRSN11709 carried two different plasmids (Table 1). Notably, MRSN7088 carried the well-characterized pRAY plasmid, a small mobilizable element frequently associated with aminoglycoside resistance (as it carries *aadB* conferring resistance to tobramycin, gentamicin and kanamycin) and recognized for its widespread distribution among various *A. baumannii* sequence types [51] as well as other *Acinetobacter* species [49].

**Fig. 7.**
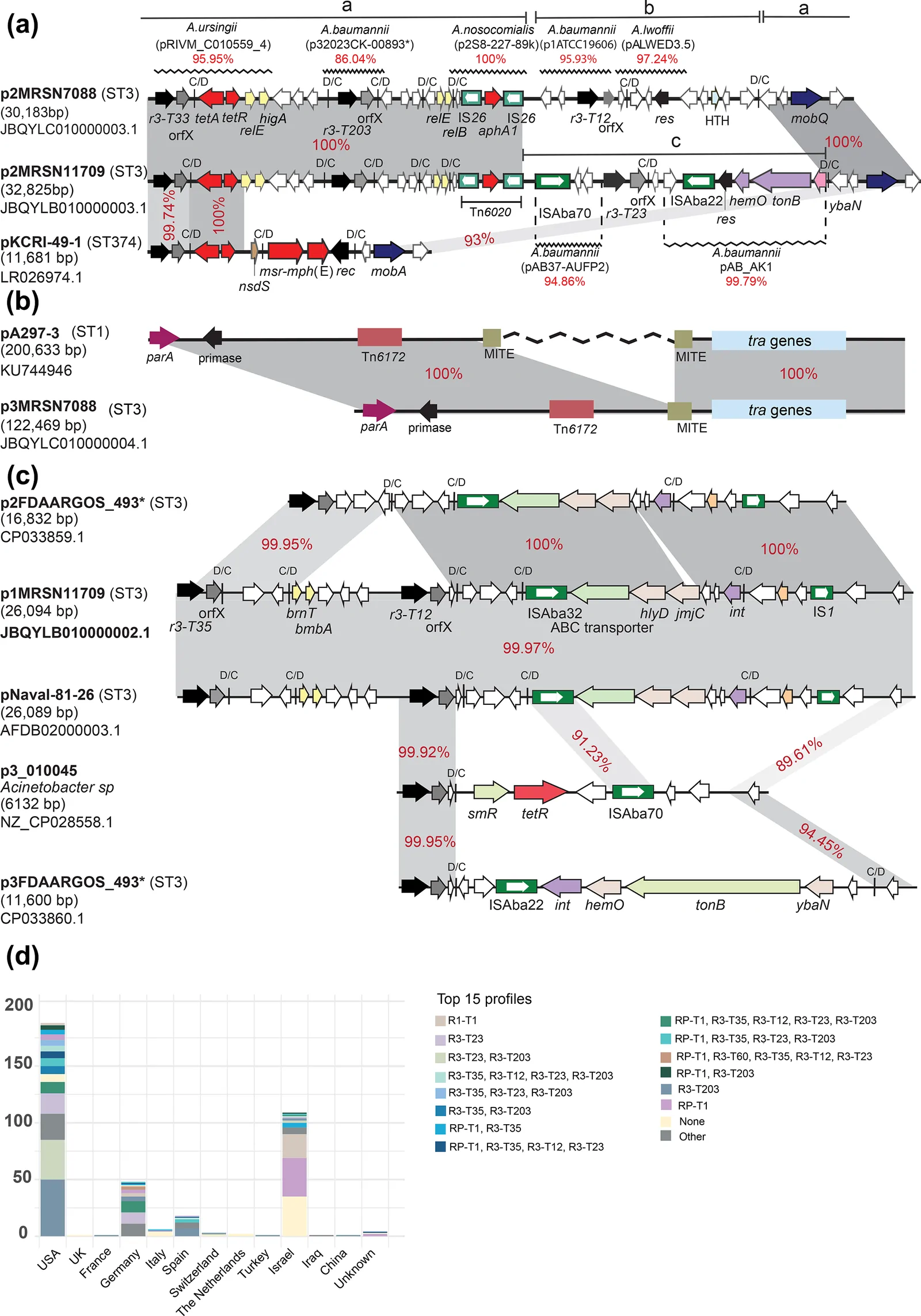
Plasmid diversity and distribution identified among ST3 and related isolates. (**a–c**) Plasmid structures in complete *A. baumannii* ST3 and related isolates. Horizontal arrows indicate genes with resistance genes shown in red. Filled green boxes indicate insertion sequences with their transposase genes indicated by arrows inside the box. Grey shading denotes shared regions with percentage identities labelled. (**d**) Distribution of top 15 plasmid Rep profiles across all countries from which ST3 isolates were recovered.

MRSN7088 contains two additional plasmids, p2MRSN7088 (30 kbp) and p3MRSN7088 (122 kbp) (Table 1, Fig. 7a and 7b). p2MRSN7088 is an R3-type plasmid and notably encodes three R3-type replication proteins (T33, T12 and T203). R3-type plasmids constitute a widely distributed replicon lineage within the genus *Acinetobacter* [33, 49, 50]. Their presence across 29 additional species highlights their broad dissemination within the genus [49]. These plasmids are characterized by a conserved *rep* gene encoding an R3-type Rep protein (i.e. Rep_3; Pfam01051) and a complex backbone structure. Several variants contain a modular structure composed of discrete *dif* modules, defined as DNA segments flanked by XerC/XerD recombination binding sites (i.e. p*dif* sites) [33, 49, 50]. Plasmid p2MRSN7088 comprises seven *dif* modules and carries the tetracycline resistance gene *tetA(A*) and the kanamycin resistance gene *aphA1* (Fig. 7a). The region containing *tetA*(A) appears to have originated from Tn*1720* and was subsequently incorporated into a *dif* module. Notably, part of this *tetA*(A)-containing module was also identified in two other R3-type plasmids (ST374), although within different *dif* modules, suggesting that *tetA*(A) has been independently acquired by distinct *dif* modules on multiple occasions. The *aphA1* gene is in Tn*6020* within a *dif* module (Fig. 7). Plasmid p3MRSN7088 was identical to pA297-3 (GenBank accession no. KU744946.1), except for the absence of the segment flanked by two identical miniature inverted-repeat transposable elements (MITEs) (Fig. 7b). This deletion likely arose from recombination between the two MITE sequences. pA297-3 is representative of a diverse group of large conjugative plasmids that contribute to the dissemination of various ARGs, including those conferring carbapenem resistance [49, 52].

MRSN11709 harbours two R3-type plasmids, p1MRSN11709 (26 kbp) and p2MRSN11709 (32 kbp) (Fig. 7a, c). p2MRSN11709 shares a substantial segment with p2MRSN7088 (labelled ‘a’ in Fig. 7a) but differs in the regions designated ‘b’ and ‘c’. These differences likely arose through multiple module exchange or acquisition events, together with IS*26*-mediated adjacent deletions (Fig. 7a). Different segments of p2MRSN11709 and p2MRSN7088 were tracked and found in distantly related plasmids in three different *Acinetobacter* species, indicating a complex evolutionary history and exchange within the genus (Fig. 7a).

Given the central role of *Acinetobacter* plasmids in the dissemination of genetic material, we subsequently typed all plasmid Rep sequences within the ST3 dataset to determine the plasmid replicon types associated with this lineage (Fig. 7d). Twenty Rep-types were observed across the entire set (*n*=383), including 14 R3-types (T1, T2, T3, T4, T8, T12, T13, T19, T23, T33, T35, T52, T60 and T203), R1-T1 and RP-T1 (Fig. 7d). R3-type plasmids are common in *Acinetobacter*; however, while T35, T60 and T203 have previously been observed in *A. baumannii*, T12 and T23 had been mainly found in non-*baumannii Acinetobacter* plasmids, including *Acinetobacter variabilis* and *Acinetobacter wuhouensis* [49]. R1-T1 is the most common R1-type sequence and is generally associated with cryptic plasmids [49]. RP-T1 is the most common RP-type sequence, encoded by conjugative plasmids that frequently carry aminoglycoside and carbapenem resistance genes [49]. We also studied Rep’s co-occurrence across the dataset, and in total, 71 unique profiles were identified including 9 profiles with only a single Rep-type. This included both geographically shared and unique profiles (Fig. 7d), indicating numerous local plasmid acquisition events.

### Distribution of virulence-associated genes

To examine virulence potential, all ST3 genomes were screened for virulence determinants that are commonly detected in *A. baumannii*. These included several known genes involved in key cellular functions such as iron acquisition, biofilm formation, capsule biosynthesis and regulatory processes as previously reported [34]. Of the 29 virulence loci that were screened, all but two (*blp,* biofilm-associated protein and *tfpO,* involved in pilin glycosylation) were detected in most ST3 genomes (Table 4). Although experimental validation is required to determine the virulence potential of ST3 genomes, lack of *blp* and *tfpO* suggests that ST3 strains are likely to form biofilm with reduced adherence capacity as previously reported [53]. This could potentially lead to decreased persistence within the host and may result in infections that are less persistent or more readily cleared compared to strains with stronger biofilm-forming capabilities.

**Table 4.** Distribution of virulence-associated determinants

Virulence	Protein acc. no.*	MRSN7088	MRSN11709	Ab238	Ab248	FDAARGOS_493
**Outer membrane proteins**						
*blc*	AKA30801.1	+	+	+	+	+
*ompA*	AKA32295.1	+	+	+	+	+
*smpA*	AKA32747.1	+	+	+	+	+
**Biofilm formation**						
*csuA*	AKA31228.1	+	+	+	+	+
*csuB*	AKA31229.1	+	+	+	+	+
*csuC*	AKA31230.1	+	+	+	+	+
*pgaA*	AKA31295.1	+	+	+	+	+
*pgaB*	AKA31296.1	+	+	+	+	+
*pgaC*	AKA31297.1	+	+	+	+	+
*pgaD*	AKA31298.1	+	+	+	+	+
*bap*	AKA30645.1	+	+	+	+	+
*blp*	AKA30673.1	−	−	−	−	−
**Capsule biosynthesis**						
*wzc*	AKA33503.1	+	+	+	+	+
*galU*	AKA33489.1	+	+	+	+	+
*wzi*	AKA32612.1	+	+	+	+	+
**Protein and pilin glycosylation**						
*pglL*	AKA30091.1	+	+	+	+	+
*tfpO*	WP_000914629.1	−	−	−	−	−
**Siderophore**						
ABUW_2189	AKA31917.1	+	+	+	+	+
ABUW_2188	AKA31916.1	+	+	+	+	+
ABUW_2187	AKA31915.1	+	+	+	+	+
ABUW_2186	AKA31914.1	+	+	+	+	+
ABUW_2185	AKA31913.1	+	+	+	+	+
ABUW_2184	AKA31912.1	+	+	+	+	+
ABUW_2183	AKA31911.1	+	+	+	+	+
*bauA*	AKA30928.1	+	+	+	+	+
**Regulatory proteins**						
*bmfR*	AKA32888.1	+	+	+	+	+
*bmfS*	AKA32887.1	+	+	+	+	+
*gigA*	AKA32956.1	+	+	+	+	+
*gacA/S*	AKA33198.1	+	+	+	+	+

*Locus IDs are based on the whole-genome sequence of *A. baumannii* strain AB5075-UW (GenBank no. CP008706.1).

## Conclusions

Despite its broad geographical distribution and evidence of long-term evolution since its apparent emergence in 1958, ST3 has not evolved as a highly successful global clone. While ST3 genomes retain key virulence determinants, only a subset has acquired carbapenem resistance genes (i.e. *bla*_OXA-23_ and *bla*_OXA-24_), and their overall ARG repertoire remains relatively limited compared with dominant clones such as ST1 and ST2. Extensive recombination, including large chromosomal exchanges and the acquisition of resistance islands such as AbST3GRI, has contributed to genomic diversification but has not resulted in sustained global expansion. Together, our findings suggest that, despite ongoing genetic exchange and diversification, ST3 lacks the combination of traits required for widespread global success.

## Supplementary material

10.1099/mgen.0.001819 Table S1.

10.1099/mgen.0.001819 Table S2.

## Abbreviations

AbST3GRI: *A. baumannii* ST3 genomic resistance island
ARG: antibiotic resistance gene
cgMLST: core genome Multi-Locus Sequence Type
ECIII: European Clone III
MITE: miniature inverted-repeat transposable elements
ML: maximum-likelihood
MLST: multi-locus sequence typing
MRSN: Multidrug-Resistant Organism Repository and Surveillance Network
ST3: sequence type 3
