# Supplementary material for: The (un)successful global spread of Acinetobacter baumannii ST3
Source: Microb Genom. 2026 Sep 15;12(9):001819. doi: 10.1099/mgen.0.001819 (PMC13583062; doi:10.1099/mgen.0.001819)
Supplement: Supplementary file 1 [file mgen-12-01819-s002.pdf]

Table S2. Prevalence of ST3 Compared to Dominant Sequence Types by Country

Country	Total number of genomes	Top Three STs (#s)	Number of ST3 Genomes (%)
USA	17060	ST2 (11184), ST499 (2010), ST406 (397)	186 (1.11%)
China	9110	ST2 (7698), ST164 (231), ST40 (125)	2 (0.02%)
Germany	1407	ST2 (349), ST1 (88), ST19 (85)	48 (3.43%)
France	520	ST25 (136), ST78 (40), ST1 (31)	1 (0.19%)
Italy	368	ST2 (336), ST3 (6), ST22 (2)	6 (1.63%)
Spain	318	ST2 (167), ST3 (18), ST18 (18)	18 (5.66%)
Israel	307	ST2 (112), ST3 (109), ST103 (35)	109 (35.50%)
Switzerland	259	ST2 (111), ST1 (17) ST 78 (10), ST353 (10)	2 (0.77%)
United Kingdom	142	ST404 (42), ST2 (31), ST162 (14)	1 (0.70%)
The Netherlands	101	ST2 (44), ST1(16), ST32(6)	2 (4.55%)
Turkey	71	ST2 (51), ST604 (6), ST1(2)	1 (1.41%)
Iraq	57	ST1090 (18), ST1(12), ST2 (9)	1 (1.75%)
